# Games of Skill

**Published:** 1869-08

**Authors:** 


					﻿SABBATH DAYS.
Many a man has confessed under the gallows, that his down-
ward progress began with misspent Sabbaths. Upon investiga-
tion, it will be often found, that the first steps taken were in what
many call “ innocent recreations,” taking a drive, wandering in
fields, loiterings by the river side, or visiting neighbors.
At home, or at church, are the places for spending the hours
of the sacred day; especially is it the way of safety for young
people—safety from the grog-shop, the engine-house, and the
chambers of her whose ways go down to death: and how much
of bodily disease are traceable directly to these three places, to
say nothing of moral corruptions, any city physician, of even
moderate practice, has daily cognizance.
One of the ways of saving persons from these calamities is, to
offer facilities for spending at least some of the hours of the
Sabbath in religious worship. To make this practical in a
single point of view, we state an experience of our own within
a month: The afternoon service by our own minister being
necessarily omitted, we went to the next church in the same
street at the usual hour of half past three o’clock. The bell
was ringing, the church doors were open, but beyond one old
lady taking her seat, there was not a living creature to be seen.
The bell tolled on. In the course of a quarter of an hour single
individuals began to drop in. , We made inquiries of several as
to the hour of service; we had asked near a dozen persons, not
one of whom knew anything. The questions came pouring in
upon us: Who preaches here ? Is there any service this after-
noon? What time does church call? Is this an Episcopal
Church? Soon there was a crowd of well-dressed men and
women standing about the door, looking in, and looking around.
At last, persons began to come, who, by their direct passing on,
seemed to be at home; but neither volunteering information or
a seat to any of the standing company; the crowd increased,
and the bell tolled on. As near half an hour is a long time for
a professional man in a large city to stand with his finger in his
mouth, we concluded we would pass on to some more hospitable
vestibule, and visited two churches in succession, both of which
were closed, to wit, on the third day of May.
The derelict church occupied a frontage of some two hundred
feet on Fifth Avenue.
The practical thought occurred to us as the people came and
went away, that for the sum of two dollars, a neat frame might
have been placed against the wall in the vestibule, stating, first,
the hours of service, and then designating some portion of the
building where strangers might seat themselves, without waiting
for the sexton to get through his half hour’s bell-tolling.
We might further suggest, that a “ lot ” be sold off, the interest
on the proceeds of which should be appropriated annually to
the hire of two active young men, whose business it should be,
while the sexton was tugging at the bell-rope, to answer the
inquiries of strangers, and courteously show seats to such as
wanted them. We may also add, and that too from our own
experience as a world’s traveller, our conviction of the great
convenience it would be to travellers to see in the hotels a
kind of church directory, in a neat frame, near the clerk’s desk
or registry-book, stating where, and at what hours and days,
religious worship was held. Each of the half dozen principal
denominations could have a separate column, all in a frame of
a single square foot. We once walked the streets-of London
for a full hour on a beautiful Sunday morning in search of a
Methodist or Presbyterian congregation, asked every police-
officer we saw, without accomplishing our object.
We know very well, that in any of our large cities, there is
not a night or a Sabbath day which does not find many strangers
who would very gladly avail themselves of the opportunity of
listening to some favorite or celebrated clergyman, if they knew
where and when to go. Inexperienced persons may say: “It is
easy to ask the clerk.” That might answer in a small town: but
in -a city, it would be a hopeless work. In our large hotels, espe-
cially in Broadway, there is only one question that is either defi-
nitely, courteously, or correctly answered; and that is, the amount
of your bill. New York physicians know, by experience, the
difficulty of finding a person who has sent for them profession-
ally. The flash hotel of Broadway, where people from the
country will “ put up ” at, for the sake of “ having it said ” they
stopped there, is notorious for this inattention. We have no
idea that there is any malice in it, but simply indifference; they
know nobody, except by the number of the room occupied:
190	Hall's Journal of Health.
General Scott is number “ twenty,” and Tom Thumb is number
“ twenty-two,” and he is most regarded who has the most
extras. These things, ought not to be, but they are; and we
adduce them as illustrations of the policy of having a church
directory in an ornamental frame, hung up in each of the pro-
minent hotels of our cities. We believe its practical effect
would be to save many persons, in the course of a year, from
falling into temptation and a snare, and disreputable disease.
				

